# Microbiological Quality and Safety of Fresh Rabbit Meat with Special Reference to Methicillin-Resistant *S. aureus* (MRSA) and ESBL-Producing *E. coli*

**DOI:** 10.3390/antibiotics13030256

**Published:** 2024-03-13

**Authors:** Jessica da Silva Guedes, David Velilla-Rodriguez, Elena González-Fandos

**Affiliations:** Food Technology Department, CIVA Research Center, University of La Rioja, Madre de Dios 53, 26006 Logrono, Spain

**Keywords:** food safety, meat safety, antimicrobial resistance, foodborne pathogens, public health, staphylococci, *E. coli*, coagulase negative staphylococci, coagulase positive staphylococci, *Mammaliicoccus*

## Abstract

The purpose of this investigation was to evaluate the microbial quality and safety of rabbit meat. A total of 49 rabbit meat samples were taken at the retail level. The mesophiles, staphylococci, *Enterobacterales*, and *Pseudomonas* spp. counts were 4.94 ± 1.08, 2.59 ± 0.70, 2.82 ± 0.67, and 3.23 ± 0.76 log CFU/g, respectively. *Campylobacter* spp. were not detected in any sample. *Listeria monocytogenes* was isolated from one sample (2.04%) at levels below 1.00 log CFU/g. Multi-resistant *S aureus* was found in seven samples (14.9%). Methicillin-resistant *S. aureus*, *S. epidermidis*, *S. haemolyticus*, *M. caseolyticus*, and *M. sciuri* were found in a sample each (10.20%), and all of them were multi-resistant. Multi-resistant ESBL-producing *E. coli* were detected in two samples from the same retailer (4.08%). The high resistance found in methicillin-resistant staphylococci and ESBL-producing *E. coli* is of particular concern, and suggests that special measures should be taken in rabbit meat.

## 1. Introduction

Spain, with a production of 40,929 tons in 2022, is the largest producer of rabbit meat in the European Union, followed by France and Italy [1]. In Spain, rabbit meat represents the fifth most consumed type of meat after pigs, poultry, cattle, sheep, and goats [1]. At the European Union level, it should be noted that there are few producing countries, since the consumption of rabbit meat is linked to cultural factors [1]. However, rabbit meat is considered as one of the healthiest types of meat due to its low fat content, high percentage of unsaturated fatty acids, low cholesterol content, high content in easily digestible protein, and B vitamins and minerals contents (mainly calcium, magnesium, and zinc) [2].

Rabbit meat is a highly nutritious substrate with a high water activity (0.99) suitable for the growth of most microorganisms [3]. Rabbit meat contamination can occur during the production process, including slaughtering and storage [3]. Contamination can originate from the animal, environment, equipment, or workers [3]. The bacteria associated with the spoilage of rabbit meat are mainly *Pseudomonas*, lactic acid bacteria, and *Brochothrix thermosphacta* [4,5,6]. Few studies have been focused on the identification of the microbiota present in rabbit meat [7]. 

Pathogens found in rabbit meat include *Salmonella*, *Escherichia coli*, *Staphylococcus aureus*, *Yersinia enterocilotica*, and *Listeria monocytogenes* [8,9,10,11]. Among foodstuffs, meat is the most frequently associated with foodborne outbreaks [12]. While other types of meat (poultry, pork, and beef) have been involved in foodborne outbreaks of *Campylobacter* spp., *Salmonella*, *S. aureus*, *E. coli*, and *L. monocytogenes*, data on rabbit meat are not available [12].

Today, the increase in antimicrobial resistance is considered to be a major threat to human and animal health [13]. This threat should be approached from a “One Health” perspective, considering veterinary medicine, human medicine, and the environment, since they are interconnected [14]. Therefore, a reduction in the transmission and spread of antibiotic resistance in one of these sectors may affect others [15].

The antimicrobial resistance of bacteria species from food-producing animals could affect human health, as they are potential sources of transmission to humans [16]. In fact, there is a serious concern about antimicrobial resistance bacteria present in meat, specifically, extended-spectrum-β-lactamase (ESBL)-producing *E. coli* and methicillin-resistant *S. aureus* (MRSA) [17,18]. Nevertheless, studies carried out on rabbit meat are scarce and mainly focus on the antimicrobial resistance of *E. coli*, and *S. aureus* [19,20,21,22]. On the other hand, other methicillin-resistant staphylococci (MRS) have been found in meat [23]. Recently, staphylococcal species belonging to the *S. sciuri* group (*S. sciuri*, *S. stepanovicii*, *S. lentus*, *S. vitulinus*, and *S. fleurettii*) were reassigned to the genus *Mammaliicoccus* [24]. Consequently, it is also important to evaluate the prevalence of MRS and methicillin-resistant *Mammaliicoccus* (MRM) in rabbit meat.

The aim of this work was to study the microbiological quality and safety of rabbit meat, together with the prevalence of methicillin-resistant *S. aureus*, other methicillin-resistant staphylococci, methicillin-resistant *Mammaliicoccus*, and ESBL-producing *E. coli*.

## 2. Results

### 2.1. Microbiological Quality and Safety of Rabbit Meat

The microbial counts of the 49 rabbit meat samples analysed are shown in Table 1. Mesophiles counts varied between 1.90 and 7.59 log CFU/g, with an average of 4.94 ± 1.08. Mesophiles levels above 7 log CFU/g were obtained only in two samples from the retailer SG (2.04%). Table 1 shows the microbial counts obtained in samples from different retailers. No significant differences (*p* > 0.05) in mesophiles counts were observed among the samples from different types of retailers or from the same type of retailer (traditional shops, supermarkets, or hypermarkets) (Table 2).

The bacteria isolated from Plate count agar in samples from hypermarkets were mainly lactic acid bacteria (30.24%), followed by *Micrococcacceae* (18.62%) *Brochotrix thermosphacta* (13.95%), *Pseudomonas* spp. (11.63%), and *Enterobacterales* (9.31%). In samples from supermarkets, the predominant bacteria were *Pseudomonas* spp. (35.48%), followed by lactic acid bacteria (26.24%), *Brochotrix thermosphacta* (9.93%), *Micrococcacceae* (7.81%), and *Enterobacterales* (5.681%), while in samples from traditional shops, the predominant bacteria were *Pseudomonas* spp. (37.50%) and *Micrococcaceae* (37.50%) (Table 3).

Staphylococci counts below 1 log CFU/g were found in 11 rabbit meat samples (22.45%). The other 38 samples showed counts ranging between 1.30 and 4.13 log CFU/g, with an average number of 2.59 ± 0.70 (Table 1). No significant differences (*p* > 0.05) in staphylococci counts were observed among samples from different types of retailers or from the same type of retailer (traditional shops, supermarkets, or hypermarkets) (Table 2). 

Table 4 shows the *Staphylococcus* spp., *Mammaliicoccus* spp., and *Macrococcus* spp. distribution, with *M. vitulinus*, *S. equorum*, and *S. saprophyticus* being the dominant species in the samples from hypermarkets, supermarkets, and traditional shops, respectively. *S aureus* was detected in one sample of hypermarket HB and four samples from supermarkets (two from supermarket SC and two from supermarket SG).

Methicillin-resistant strains were found in five samples when using chromID MRSA agar, with one from hypermarket HA being identified as *S. epidermidis* and four from supermarkets being identified as *S. aureus* (SC), *S. haemolyticus* (SF), *M. sciuri* (SD), and *M. caseolyticus* (SH).

*Enterobacterales* counts below 1 log CFU/g were found in 17 samples (34.69%). The counts in the other 32 samples ranged between 1.30 and 4.74 CFU/g, with an average number of 2.82 ± 0.67 (Table 1). No significant differences (*p* > 0.05) in *Enterobacterales* counts were found among rabbit samples from different types of retailers or from the same type of retailer (Table 2). Table 5 shows the species distribution. *Serratia liquefaciens* was the dominant species in samples from supermarkets and traditional shops, while *Ewingella americana* was the predominant species in samples from hypermarkets. *E. coli* was found in samples from hypermarkets and supermarkets. When using ChromID ESBL, *E. coli* was found in two samples from supermarket SF.

*Pseudomonas* spp. counts below 1 log CFU/g were found in 14 samples (28.57%). The other 35 samples (71.43%) showed counts between 1.30 and 6.11 log CFU/g, with an average number of 3.23 ± 0.76 (Table 1). Significant differences (*p* < 0.05) in pseudomonas counts were observed among samples from different supermarkets (Table 2). Table 6 shows the *Pseudomonas* spp. distribution, with *P. libanensis* and *P. extremorientalis* being the dominant species in the samples from hypermarkets and supermarkets, while the dominant species in samples from traditional shops were *P. fluorescens* and *P. libanensis*.

*Campylobacter* spp. were not detected in any sample. *L. monocytogenes* was only found in one sample from supermarket SG at levels below 1 log CFU/g. *L. innocua* was isolated from two samples, one from supermarket SG and one from a traditional shop (TJ).

### 2.2. Antimicrobial Resistance

The antimicrobial resistance phenotype of 96 strains of *Staphylococcus* spp., *Mammaliicoccus* spp., and *Macrococcus* spp. isolated from rabbit meat was evaluated. In total, 68 strains were resistant to 1 or more antibiotics (70.83%), of which 24 were multi-resistant (resistant to ≥3 different classes of antibiotics) (25%). Considering *S. aureus*, 80% of the strains showed multi-resistance, 20% being methicillin-resistant (MRSA). A total of 80% of *S. aureus* strains were resistant to ciprofloxacin and enrofloxacin, 70% were resistant to kanamycin, tobramycin, erythromycin, and lincomycin, 60% were resistant to gatifloxacin, levofloxacin, gentamicin, streptomycin, and clindamycin, and 50% were resistant to norfloxacin. In addition, 40% of the strains were resistant to benzilpenicillin, tetracycline, and penicillin, 30% were resistant to doxycycline, 20% were resistant to tylosin, and 10% were resistant to quinupristin/dalfopristine, amikacin, mupirocin, and trimethoprim. None of the *S. aureus* strains were resistant to fusidic acid, ceftaroline, chloramphenicol, linezolid, trimethoprim-sulfamethoxazole, vancomycin, or ampicillin. 

Regarding the coagulase-negative staphylococci and *M. caseolyticus* strains, 19.77% were multi-resistant and 8.14% were methicillin-resistant. The highest resistance rates were found against lincomycin, tetracycline, and penicillin (24.42%), fusidic acid (20.93%), followed by erythromycin (16.28%), mupirocin (12.79%), clindamycin (11.63%), doxycycline (10.47%), streptomycin (8.14%), cefoxytin (8.14%), enrofloxacin (4.65%), kanamycin (4.65%), and sulfadiazine (4.65%). Lower resistance rates were observed against amikacin, ciprofloxacin, levofloxacin, tobramycin (3.49%), nitrofurantoin, rifampicin, tedizolid (2.33%), norfloxacin, and tylosin (1.16%). None of the isolates were resistant to ceftaroline, chloramphenicol, gentamycin, gatifloxacin, linezolid, trimethoprim, trimethoprim-sulfamethoxazole, vancomycin, or ampicillin.

The phenotype of the multi-resistant strains is shown in Table 7. Eight strains were resistant to methicillin, seven from supermarkets (SC, SD, SF, and SG) and one from hypermarket HA. Multi-resistant *S. aureus* strains were found in samples from supermarkets SC and SG, being resistant to ≥8 antibiotics. Multi-resistant strains of other species were found with a higher prevalence in supermarket SF and hypermarket HA. Strains of *M. caseolyticus*, *S. haemolyticus*, and *S. lugdunensis* showed resistance against 14, 12, and 10 antibiotics, respectively. Multi-resistant *S. epidermidis* strains were detected in hypermarket HA and supermarket SF. Most of the multi-resistant strains were isolated from supermarkets (19 strains), followed by hypermarkets (4 strains from HA), and 1 strain from a traditional shop (TI).

The antimicrobial resistance phenotype of 12 strains of *E. coli* isolated from rabbit meat was evaluated (Table 8). All the strains were multi-resistant. The strains isolated from ChromID ESBL were phenotypically confirmed as ESBL-producing, and the other *E. coli* strains were not ESBL-producing. Most of the multi-resistant strains were isolated from supermarkets (10 strains), followed by hypermarkets (2 strains from HB). The highest resistance rates were observed for streptomycin and tetracycline (100.00%), followed by doxycycline and sulfadiazine (91.67%), sulfamethoxazole-trimethoprim and trimethoprim (83.33%), and colistin (66.67%). The resistance rates for quinolones were 41.67% for norfloxacin, levofloxacin, and gatifloxacin, 66.67% for ciprofloxacin, and 75.00% for nalidixic acid and enrofloxacin. Four strains were resistant to cefpodoxime (33.33%). In the penicillin group, the highest resistance rates were observed against ampicillin and piperacillin (25.00%). In total, 25% of the strains were resistant to aztreonam, ceftazidime, ceftriaxone, chloramphenicol, ertapenem, and tobramycin. Two strains (16.67%) showed resistance to the antibiotics cefoxitin, meropenem, and tigecycline. In addition, one strain (8.33%) presented resistance against amikacin, ampicillin with sulbactam, cefepime, cefotaxime, doripenem, and imipenem. All strains were sensitive to ampicillin, amoxicillin-clavulanate, kanamycin, and nitrofurantoin.

## 3. Discussion

In the present study, mesophiles counts varied between 3.79 ± 0.96 and 6.06 ± 1.02, depending on the retailer where the rabbit samples were purchased. Considering the 49 samples analysed, the average was 4.94 ± 1.08. These results are consistent with those reported by other authors. Thus, Cwiková and Pytel reported mesophiles counts of 5.34 log CFU/g. Similar counts were found by Wang et al. (2021) (4.56 log CFU/g) [25]. Rodríguez-Calleja et al. evaluated rabbit meat from two different supermarkets, obtaining mesophiles counts of 5.87 ± 1.03 and 6.60 ± 1.18 log CFU/g [5]. We found mesophiles counts above 7 log CFU/g in two samples acquired from a supermarket (SG); these levels are associated with meat spoilage [4,5]. Differences in mesophiles counts can be explained by handling, time, and storage conditions. Hygiene and the proper handling of rabbit meat are associated with low levels of contamination [4,10]. In addition, other factors such as time and storage conditions also influence meat quality [26]. In our work, significant differences were not observed among types of retailers or among the same type of retailer. Rodríguez-Calleja et al. did not find differences in the mesophiles counts between the rabbit samples of the two supermarkets evaluated either [5].

The most common bacteria isolated from rabbit meat were *Brochothrix thermosphacta*, lactic acid bacteria, and *Pseudomonas* spp., which is in accordance with the results obtained in the present study [3,6]. However, *Micrococcaceae* can be one of the dominant bacteria in rabbit meat, as shown by the results obtained in samples from traditional shops.

In the current work, *Staphylococcus* spp. counts varied between 1.66 ± 0.08 and 3.01 ± 0.23 log UFC/g, depending on the retailer where the samples were purchased. Considering the 49 samples analysed, the average was 2.59 ± 0.70 log CFU/g. Other authors have evaluated the coagulase-positive staphylococci counts of rabbit meat, obtaining values of 1.18 ± 0.44 and 2.01 ± 1.02, depending on the place of purchase [5]. We isolated *S. aureus* from rabbit samples purchased in hypermarkets and supermarkets; also, Cullere et al. detected *S. aureus* in rabbit meat [27]. Similar to Pipová et al., we observed a higher prevalence of coagulase-negative staphylocci than coagulase-positive staphylococci in rabbit meat [22]. It should be noted that some coagulase-negative staphylococci have occasionally been associated with human infections (*S. epidermidis*, *M. sciuri*, *S. cohnii*, *S. saprophyticus*, *S. simulans*, *S. hyicus*, and *S. warneri* [28,29,30,31]. In fact, *S. epidermidis*, *M. sciuri*, *S. saprophyticus*, *S. simulans*, and *S. warneri* were isolated from rabbit meat in the present work. In addition, *S. saprophyticus* was the dominant staphylococci found in samples from traditional shops. We identified 14 different species of staphylococci, while other authors have reported only 8 different species, including *S. aureus*, *S. warneri*, *S. epidermidis*, *S. pasteuri*, *S. xylosus*, *S. capitis*, *S. haemolyticus*, and *S. cohnii* [22]. We did not isolate *S. xylosus*, *S. capitis*, and *S. cohnii*. While Pipová et al. reported that the dominant species in rabbit meat were *S. warneri* (45.1%) and *S. epidermidis* (21.2%), we found that the dominant species were *M. vitulinus* (51.52%), *S. equorum* (17.89%), and *S. saprophyticus* (31.25%) in samples obtained from hypermarkets, supermarkets, and traditional shops, respectively [22].

Lower *Enterobacterales* counts in rabbit meat have been reported by other authors [4,32]. Pereira and Malfeito-Ferreira reported *Enterobacterales* counts of 1.8 ± 1.35 log CFU/g on day 0 of storage (2.82 ± 0.67 log CFU/g in the present work) [4]. Also, lower counts were reported by Koné et al., with 1.81 ± 0.10 on day 0, but after 6 days of storage, the levels increased to 4.24 ± 1.55 [32]. The differences found can be explained by the hygienic measures taken during meat processing and the storage conditions (time, temperature, and packaging) [9]. It should be highlighted that *Enterobacterales* are used like an indicator of the hygienic conditions during slaughter, because they are related to faecal contamination [33]. In this study, the dominant *Enterobacterales* species varied according to the type of retailer. *Ewingella americana*, *Serratia proteamaculans*, and *Yersinia intermedia* were the predominant species in samples from hypermarkets (23.08–30.77%), while *S. liquefaciens* was the dominant bacteria in samples from supermarkets and traditional shops (45.16 and 100%, respectively). Also, *S. liquefaciens* has been identified as the dominant bacteria in other types of meat [17].

We observed that *Pseudomonas* spp. counts in rabbit meat varied between 1.30 and 6.11 log CFU/g, with an average of 3.23 ± 0.76 log CFU/g. Lower pseudomonas counts were reported by Pereira and Malfeito-Ferreira in rabbit carcasses (2.68 ± 0.85 log CFU/g, ranging between 1.00 and 3.99 log CFU/g) [4]. The differences found can be explained by storage conditions (time, temperature, and packaging) [10,27,34]. Thus, Nakyinsige et al. reported that pseudomonas counts increase with storage time (3.44 ± 0.16 on day 0, and 5.58 ± 0.08 on day 7 of storage) [34]. Similar counts were observed by Rodriguez-Calleja et al. (3.39 ± 1.12 on day 0 of storage) [6]. It should be noted that *Pseudomonas* spp. is responsible for the deterioration of meat due to chromatic alterations related to the enzymatic activity of this bacterium [7]. In the present study, 31 *Pseudomonas* spp. were identified in rabbit meat, with *P. libanensis*, *P. extremorientalis*, and *P. fluorescens* being the dominant species. There have been few works on the identification of *Pseudomonas* spp. in rabbit meat, and they are focused on the detection of bacteria responsible for spoilage [7].

The presence of *Listeria* spp. in rabbit meat has also been reperted by other authors, although in a higher percentage (13.7% vs. 6.12% in the present work) [8]. Other authors have also isolated *L. innocua* from rabbit meat [8]. We only detected the presence of *L. monocytogenes* in 2.04% of the rabbit meat samples, lower than the values reported by other authors (7.32–38%) [8,35,36].

We did not detect *Campylobacter* spp. in any rabbit sample, which is agreement with other studies that have not detected this pathogen in rabbit farms [37,38].

We observed that 25% of the staphylococi strains were multi-resistant, and similar results have been reported by Pipová et al. [22]. In the current work, 80% of *S. aureus* strains showed multi-resistance, with 20% being methicillin-resistant (MRSA). In contrast, other authors did not detect any MRSA isolate from rabbit meat, although it was detected in other types of meat (poultry and pork) [21,39]. However, other authors have reported the presence of MRSA in rabbit meat [3,40]. Similar to Mosrhdy et al. we observed a high resitance rate against erythromycin (82.4% vs. 70% in the present work) [3]. We found higher resistant rates against ciprofloxacin (80%) and norfloxacin (50%) in *S. aureus* strains than Morshdy et al. (17.6% and 29.4%, respectively) [3]. However, these authors observed a high resistance rate against chloramphenicol (88.2%), while we observed that all the *S. aureus* strains were susceptible. We observed that 60% and 20% of the *S. aureus* strains isolated from rabbit meat were resistant to gentamicin and mupirocin. However, other authors have reported no resistance to these antibiotics among *S. aureus* strains isolated from rabbit meat [40]. Other authors have also resported that *S. aureus* strains isolated from rabbit meat were susceptible to chloramphenicol, trimethoprim/sulfamethoxazole, and fusidic acid [40]. Regarding the coagulase-negative staphylococci and *M. caseolyticus* strains, 19.77% were multi-resistant and 8.14% were methicillin-resistant. Also, Pipová et al. reported that 8% of the staphylococci isolated from rabbit meat were methicillin-resistant [22]. Higher resistance rates to erythromycin (58.4%) and penicillin (51.3%) were reported by Pipova et al. than those in the present work (16.8 and 24.42%, respectively) [22]. It should be noted that we found that 10% of the *S. aureus* strains and 12.79% of the other staphylococci were resistant to mupirocin. Moreover, 2.33% of the coagulase-negative staphylococci were resistant to rifampicin. Both mupirocin and rifampicin are classified as antimicrobials to avoid in animals “Category A” [41].

All the *E. coli* strains evaluated from the rabbit meat were multi-resistant. The presence of ESBL-producing *E. coli* was detected in 8.16% of the samples (two samples), all of them from supermarket SF (40% of the samples from supermarket SF). In contrast, Stewardson et al. did not detect ESBL-producing *E. coli* in rabbit meat [19]. Also, Kylie et al. reported high resistance rates in *E. coli* strains isolated from rabbit meat, especially against tetracycline [20]. We observed differences in multi-resistance among retailers, as they were only isolated from hypermarkets HB and all the supermarkets, except supermarket SD. The high rate of multi-resistant strains found is in accordance with those described by Martinez-Laorden et al., which found high rates of multi-resistance for *E. coli* isolated from turkey meat (71.43–100%) [17]. We found that 25% of the *E. coli* strains were resistant to aztreonam, 16.67% to meropenem, and 16.67% to tigecycline. These findings are relevant, since aztreonam, meropenem, and tigecycline are classified as antimicrobials to avoid in animals “Category A” [41].

Our results suggest that special care should be taken to avoid the contamination of rabbit meat during slaughtering and handling.

## 4. Materials and Methods

### 4.1. Rabbit Samples and Microbiological Analysis

Forty-nine rabbit meat samples were taken from hypermarkets (HA, HB), supermarkets (SC, SD, SE, SF, SG, SH), and traditional shops (TI, TJ) in Logroño (Spain). The number of samples taken at each sale point was established according to the trade model and their readiness [42]. Rabbit meat samples were brought to university facilities in refrigerated conditions and analysed within two hours. For analysis, 10 g from the legs was aseptically taken and homogenized, as described by Silva et al. [43]. The following analyses were conducted: mesophiles, staphylococci *Enterobacterales*, *Pseudomonas* spp., *Campylobacter* spp., and *Listeria* spp., as described by Silva et al. [43]. Also, the analysis of methicillin-resistant *S. aureus* (MRSA) and ESBL-producing *E. coli* was performed as described by Silva et al. [43]. Table 9 shows the conditions used for the microbiological determinations. 

The presence of Campylobacter spp. and *L. monocytogenes* in rabbit meat samples was determined as described by Da Silva et al. [43].

### 4.2. Isolation and Identification 

From each culture media and sample, between three and five colonies were randomly selected. The appearance of suspected colonies was considered when selective media were employed. Tryptone Soy Agar (Scharlau) was used to purify isolates. The isolates were maintained at −80 °C. Bacterial identification was performed by a Matrix-Assisted Laser Desorption/Ionization-Time of Flight Mass-Spectrometry (MALDITOF MS) Biotyper (Bruker, Billerica, MA, USA).

### 4.3. Confirmation of Methicillin Resistance of Mammaliicoccus spp. and Staphylococcus spp.

The methicillin resistance of *Mammaliicoccus* spp. and *Staphylococcus* spp. obtained from ChromID MRSA agar, and all the *S. aureus* strains obtained, was confirmed following the criteria described in the Clinical Laboratory Standards Institute’s guidelines [44].

### 4.4. Phenotypic Antimicrobial Resistance of Methicillin Resistance Mammaliicoccus spp. and Staphylococcus spp.

The resistance of *Mammaliicoccus* and staphylococci was evaluated against twenty-nine antibiotics employing the disk-diffusion technique on Mueller–Hinton agar. The antimicrobials (Oxoid) used and their concentrations have been previously described [43]. The antimicrobials were: ceftaroline, cefoxitin penicillin, fusidic acid, clindamycin, tetracycline, minocycline, doxycycline, trimethoprim, enrofloxacin, levofloxacin, ciprofloxacin, norfloxacin, gatifloxacin, gentamicin, trimethoprim-sulfamethoxazole, streptomycin, amikacin, kanamycin, sulfadiazine, tobramycin, erythromycin, tylosin, mupirocin, lincomycin, chloramphenicol, nitrofurantoin, linezolid, rifampicin, tedizolid, and vancomycin. The inhibition zones were recorded after incubation at 37 °C for 18 to 24 h. Depending on the inhibition zones and antibiotic used, the Clinical and Laboratory Standards Institute’s guidelines classified the strain as resistant, susceptible, or intermediate (reduced susceptibility) [44].

### 4.5. Phenotypic Confirmation of ESBL-Producing E. coli

One *E. coli* strain identified by MALDI-TOF was chosen for each different medium and sample for phenotypic confirmation of ESBL. The confirmation was carried out according to the Clinical Laboratory Standards Institute’s guidelines [39].

### 4.6. Phenotypic Antimicrobial Resistance of E. coli Isolates

The resistance of *E. coli* strains was evaluated against 35 antibiotics employing the disk-diffusion technique on Mueller–Hinton agar, and the concentrations have been previously described [43]. The antimicrobials were: ceftazidime, ceftriaxone, cefoxitin, cefpodoxime, cefepime, aztreonam, cefotaxime, ampicillin, amoxicillin-clavulanate, ampicillin-surbactam, ertapenem, imipenem, meropenem, doripenem, piperacillin, trimethoprim-sulfamethoxazole, trimethoprim, chloramphenicol, sulfadiazine, tetracycline, minocycline, doxycycline, tigecycline, enrofloxacin, levofloxacin, ciprofloxacin, norfloxacin, gatifloxacin, nalidixic acid, amikacin, gentamicin kanamycin, streptomycin, tobramycin, and nitrofurantoin. The inhibition zones were recorded after incubation at 37 °C for 18 to 24 h. Depending on the inhibition zones and antibiotic used, the Clinical and Laboratory Standards Institute’s guidelines classified the strain as resistant, susceptible, or intermediate (reduced susceptibility) [44].

### 4.7. Statistical Analysis

Analysis of variance was conducted using SPSS version 26 software (IBM SPSS Statistics, Armonk, NY, USA). Tukey’s test for comparison of means was conducted using the same program. The level of significance was determined at *p* < 0.05.

## 5. Conclusions

This work shows that rabbit meat could be a source of methicillin-resistant *S. aureus*, methicillin-resistant staphylococci, and ESBL-producing *E. coli*. Moreover, resistance to critical antibiotics such as mupirocin, rifampicin, aztreonam, meropenem, and tigecycline was detected, being of special concern for consumer’s health. These findings highlight the need to take special measures in the frame of One Health. 

## Figures and Tables

**Table 1:** Microbial counts (log CFU/g) found in 49 rabbit meat samples.

Microbial Group	N ^1^ Counts < 1	N ^1^ Counts > 1	Minimum Counts	Maximum Counts	Mean	Standard Deviation
Mesophiles	0	49	1.90	7.59	4.94	1.08
Staphylococci	11	38	1.30	4.13	2.59	0.70
*Enterobacterales*	17	32	1.30	4.74	2.82	0.67
*Pseudomonas*	14	35	1.30	6.11	3.23	0.76

^1^ Number of samples.

**Table 2:** Microbial counts (log CFU/g) in rabbit meat from different retailers.

Type of Retailer	Retailer	N ^1^	Mesophiles	Staphylococci	*Enterobacterales*	*Pseudomonas*
Hypermarket	HA	7	5.39 ± 0.96 ^2a^_a_	2.88 ± 0.59 ^a^_a_	2.82 ± 0.78 ^a^_a_	4.06 ± 0.73 ^a^_a_
Hypermarket	HB	4	4.90 ± 0.87 ^a^_a_	1.88 ± 0.39 ^a^_a_	3.15 ± 0.12 ^a^_a_	2.46 ± 0.31 ^a^_a_
Supermarket	SC	6	3.79 ± 0.96 ^a^_a_	2.23 ± 0.61 ^a^_a_	2.73 ± 0.00 ^a^_a_	<1 ^a^_a_
Supermarket	SD	6	5.52 ± 0.36 ^a^_a_	2.99 ± 0.91 ^a^_a_	3.05 ± 0.23 ^a^_a_	3.28 ± 0.55 ^a^_b_
Supermarket	SE	6	4.06 ± 0.38 ^a^_a_	1.66 ± 0.08 ^a^_a_	1.94 ± 0.57 ^a^_a_	2.33 ± 0.45 ^a^_b_
Supermarket	SF	5	4.02 ± 0.79 ^a^_a_	2.15 ± 0.90 ^a^_a_	2.17 ± 0.82 ^a^_a_	2.56 ± 0.85 ^a^_b_
Supermarket	SG	5	6.06 ± 1.02 ^a^_a_	3.00 ± 0.82 ^a^_a_	3.09 ± 1.06 ^a^_c_	3.75 ± 1.05 ^a^_b_
Supermarket	SH	6	5.33 ± 0.52 ^a^_a_	2.23 ± 0.39 ^a^_a_	2.85 ± 0.29 ^a^_a_	3.48 ± 0.57 ^a^_b_
Traditional Shop	TI	2	4.31 ± 2.41 ^a^_a_	2.34 ± 1.04 ^a^_a_	4.00 ± 0.00 ^a^_a_	3.00 ± 0.40 ^a^_a_
Traditional Shop	TJ	2	5.13 ± 1.44 ^a^_a_	3.01 ± 0.23 ^a^_a_	2.68 ± 0.77 ^a^_a_	3.54 ± 0.00 ^a^_a_

^1^ Number of samples; ^2^ Average ± standard deviation. Averages in the same column sharing a superscript letter show no significant differences among the different types of retailers (*p* > 0.05). Averages in the same column sharing a subscript letter show no significant differences among the same types of retailers (*p* > 0.05).

**Table 3:** Bacteria identified in rabbit meat by type of retailer isolated from Plate Count Agar.

Type of Retailer	Microbial Group	Percentage (%)	Species	Percentage (%)
Hypermarket (HA, HB)	*Brochothrix* spp.	13.95	*Brochothrix thermosphacta*	13.95
	Lactic acid Bacteria	30.24	*Carnobacterium divergens*	23.26
			*Lactobacillus* spp.	4.65
			*Carnobacterium maltaromaticum*	2.33
	*Pseudomonas* spp.	11.63	*P. fragi*	6.98
			*P. libanensis*	4.65
	*Enterobacterales*		*Serratia proteamaculans*	4.65
		9.31	*Serratia liquefaciens*	2.33
			*Rahnella inusitata*	2.33
	*Micrococcaceae*	18.62	*Staphylococcus equorum*	4.65
			*Mammaliicoccus fleurettii*	4.65
			*Staphylococcus epidermidis*	2.33
			*Staphylococcus haemolyticus*	2.33
			*Staphylococcus warneri*	2.33
			*Kocuria rhizophila*	2.33
	Other Gram-positive bacteria	2.33	*Rothia endophytica*	2.33
	Other Gram-negative bacteria	13.98	*Acinetobacter albensis*	2.33
			*Acinetobacter harbinensis*	2.33
			*Chryseobacterium piscium*	2.33
			*Chryseobacterium vrystaatense*	2.33
			*Sphingobacterium faecium*	2.33
			*Stenotrophomonas maltophilia*	2.33
Supermarket (SC, SD, SE, SF, SG, SH)	*Brochothrix* spp.	9.93	*Brochothrix thermosphacta*	9.93
	Lactic acid bacteria		*Carnobacterium divergens*	12.06
		26.24	*Carnobacterium maltaromaticum*	8.51
			*Lactobacillus* spp.	5.67
	*Pseudomonas* spp.	35.48	*P. fragi*	11.35
			*P. libanensis*	5.67
			*P. extremorientalis*	4.26
			*P. fluorescens*	3.55
			*P. brenneri*	2.13
			*P. lundensis*	2.13
			*P. proteolítica*	2.13
			*P. chlororaphis*	1.42
			*P. koreensis*	1.42
			*P. azotoformans*	0.71
			*P. tolaasii*	0.71
	*Enterobacterales*		*Serratia liquefaciens*	2.13
			*Serratia proteamaculans*	2.13
		5.68	*Escherichia coli*	0.71
			*Serratia fonticola*	0.71
	*Micrococcaceae*		*Staphylococcus saprophyticus*	2.13
			*Mammaliicoccus vitulinus*	2.13
		7.81	*Mammaliicoccus fleurettii*	1.42
			*Kocuria rhizophila*	0.71
			*Staphylococcus aureus*	0.71
			*Mammaliicoccus sciuri*	0.71
	Other Gram-positive bacteria	0.71	*Arthrobacter stackebrandtii*	0.71
	Other Gram-negative bacteria	14.20	*Chryseobacterium scophthalmum*	4.26
			*Acinetobacter harbinensis*	2.84
			*Stenotrophomonas maltophilia*	1.42
			*Acinetobacter guillouiae*	0.71
			*Bordetella hinzii*	0.71
			*Chryseobacterium indoltheticum*	0.71
			*Microbacterium aurum*	0.71
			*Microbacterium paraoxydans*	0.71
			*Pantoea agglomerans*	0.71
			*Psychrobacter maritimus*	0.71
			*Stenotrophomonas* spp.	0.71
Traditional shop (TI, TJ)	*Pseudomonas* spp.	37.50	*P. fluorescens*	12.50
			*P. fragi*	12.50
			*P. lundensis*	12.50
	*Micrococcaceae*	37.50	*Staphylococcus saprophyticus*	37.50
	Other Gram-positive bacteria	25.00	*Rothia endophytica*	25.00

**Table 4:** *Staphylococcus* spp., *Mammaliicoccus* spp., and *Macrococcus* spp. isolated from rabbit meat by type of retailer (recovered from MSA).

Type of Retailer	Species	Percentage (%)
Hypermarket (HA, HB)	*Mammaliicoccus vitulinus*	51.52
	*Mammaliicoccus fleurettii*	21.21
	*Staphylococcus pasteuri*	9.09
	*Staphylococcus warneri*	6.06
	*Staphylococcus aureus*	3.03
	*Staphylococcus capitis*	3.03
	*Staphylococcus epidermidis*	3.03
	*Staphylococcus equorum*	3.03
Supermarket (SC, SD, SE, SF, SG, SH)	*Staphylococcus equorum*	17.89
	*Staphylococcus saprophyticus*	15.90
	*Mammaliicoccus vitulinus*	15.79
	*Staphylococcus aureus*	11.58
	*Mammaliicoccus fleurettii*	11.58
	*Macrococcus caseolyticus*	6.32
	*Staphylococcus epidermidis*	6.32
	*Staphylococcus pasteuri*	4.21
	*Staphylococcus warneri*	4.21
	*Mammaliicoccus sciuri*	3.16
	*Staphylococcus chromogenes*	1.05
	*Staphylococcus haemolyticus*	1.5
	*Mammaliicoccus lentus*	1.05
Traditional shop (TI, TJ)	*Staphylococcus saprophyticus*	31.25
	*Mammaliicoccus fleurettii*	18.75
	*Staphylococcus equorum*	12.50
	*Mammaliicoccus lentus*	12.50
	*Mammaliicoccus sciuri*	6.25
	*Staphylococcus simulans*	6.25
	*Mammaliicoccus vitulinus*	6.25
	*Staphylococcus warneri*	6.25

**Table 5:** *Enterobacteriacceae* isolated from rabbit meat by type of retailer (recovered from McConkey agar).

Type of Retailer	Species	Percentage (%)
Hypermarket (HA, HB)	*Ewingella americana*	30.77
	*Serratia proteamaculans*	23.08
	*Yersinia intermedia*	23.08
	*Escherichia coli*	15.38
	*Serratia liquefaciens*	7.69
Supermarket (SC, SD, SE, SF, SG, SH)	*Serratia liquefaciens*	45.16
	*Hafnia alvei*	12.90
	*Escherichia coli*	8.06
	*Serratia fonticola*	8.06
	*Ewingella americana*	6.45
	*Yersinia intermedia*	6.45
	*Buttiauxella noackiae*	3.23
	*Lelliottia amnigena*	3.23
	*Pantoea agglomerans*	3.23
	*Buttiauxella gaviniae*	1.61
	*Yersinia enterocolitica*	1.61
Traditional shop (TI, TJ)	*Serratia liquefaciens*	100

**Table 6:** *Pseudomonas* spp. isolated from rabbit meat by type of retailer (recovered from specific media for Pseudomonas).

Type of Retailer	Species	Percentage (%)
Hypermarket (HA, HB)	*Pseudomonas libanensis*	36.36
	*Pseudomonas extremorientalis*	31.82
	*Pseudomonas fluorescens*	9.09
	*Pseudomonas brenneri*	4.55
	*Pseudomonas cedrina*	4.55
	*Pseudomonas rhodesiae*	4.55
	*Pseudomonas synxantha*	4.55
Supermarket (SC, SD, SE, SF, SG, SH)	*Pseudomonas libanensis*	33.33
	*Pseudomonas extremorientalis*	17.78
	*Pseudomonas fluorescens*	16.67
	*Pseudomonas antarctica*	6.67
	*Pseudomonas fragi*	4.44
	*Pseudomonas marginalis*	4.44
	*Pseudomonas azotoformans*	3.33
	*Pseudomonas koreensis*	2.22
	*Pseudomonas rhodesiae*	2.22
	*Pseudomonas synxantha*	2.22
	*Pseudomonas tolaasii*	2.22
	*Pseudomonas veronii*	2.22
	*Pseudomonas chlororaphis*	1.11
	*Pseudomonas lundensis*	1.11
Traditional shop (TI, TJ)	*Pseudomonas fluorescens*	40
	*Pseudomonas libanensis*	40
	*Pseudomonas extremorientalis*	20

**Table 7:** Antimicrobial resistance phenotype of multi-resistant *Staphylococcus* spp., *Mammaliicoccus* spp., and *Macrococcus* spp. strains isolated from rabbit meat.

Species	Retailer	Antimicrobial Resistant Phenotype ^1^
*S. aureus*	SC ^2,3^	FOX-AK-CIP-ENR-GAT-K-LEV-PUM-NOR-P-S-SUZ-TE-TOB-PNG
	SG ^3^	FOX-CIP-DO-ENR-CN-K-MY-NOR-P-S-TE-TOB-W-TY-ERY-CMN-QD-PNG
	SG	CIP-DO-ENR-GAT-CN-K-LEV-MY-P-S-TE-TOB-ERY-PNG
	SG	CIP-DO-ENR-GAT-CN-K-LEV-MY-NOR-TE-TOB-ERY-CMN
	SC	CIP-ENR-GAT-CN-K-LEV-MY-NOR-S-TOB-ERY-CMN
	SC	CIP-ENR-GAT-CN-K-LEV-MY-S-TOB-ERY-CMN
	SC	CIP-ENR-MY-P-TY-ERY-CMN-PNG
*S. epidermidis*	SF	P-SUZ-TE-TOB-ERY
	HA ^2,3^	FOX-CIP-ENR-FAD-LEV-PUM-ERY
	HA	LEV-MY-P
*S. equorum*	SF	DO-K-MY-S-TE-ERY-CMN
*S. haemolyticus*	SF ^2,3^	FOX-CIP-ENR-LEV-MY-NOR-P-S-TE-ERY-CMN
*S. lugdunensis*	HA	FOX-AK-CIP-ENR-FAD-K-PUM-F-P-S-SUZ-TE-PNG
*S. pasteuri*	SD ^3^	FOX-PUM-P-ERY
*S. pasteuri*	SG ^3^	FOX-PUM-P-ERY
*S. saprophyticus*	SF	DO-FAD-TZD-CMN
	SF	DO-RD-TZD-CMN
*S. simulans*	TI	MY-P-ERY-CMN
*M. caseolyticus*	SH ^2,3^	FOX-AK-ENR-GAT-K-MY-MH-P-S-SUZ-TE-TOB-TY-ERY-CMN
	SG	ENR-S-TE-ERY
*M. fleurettii*	SC	FAD-MY-P-CMN
	HA	FAD-MY-P
*M. sciuri*	SD ^2,3^	FOX-AK-K-MY-PUM-S-SUZ-TE-CMN
	SE	DO-FAD-MY-S-TE-TOB

^1^ FOX: cefoxitin, AK: amikacin, CIP: ciprofloxacin, DO: doxycline, ENR: enrofloxacin, FAD: fusídic acid, GAT: gatifloxacin, CN: gentamicyn, K: kanamicyn, LEV: levofloxacin, MY: lincomycin, MH: minocycline, PUM: mupirocin, F: nitrofurantoin, NOR: norfloxacin, P: penicillin, RD: rifampicin, S: streptomycin, SUZ: sulfadiazine, TZD: tedizolid, TE: tetracicline, TOB: tobramycin, W: trimethoprim, TY: tylosin, ERY: erythromycin, CMN: clindamycin, QD: quinupristin-dalfopristin, and PNG: Benzilpenicilin. ^2^ Strain isolated from chromID MRSA, ^3^ methicillin-resistant strain.

**Table 8:** Antimicrobial resistance phenotype of *Escherichia coli* strains isolated from rabbit meat.

Retailer (Number of Isolates)	Antimicrobial Resistant Phenotype ^1^
SF (1)	AK-ATM-FEP-CTX-FOX-CPD-CAZ-CRO-C-CIP-CT-DOR-DO-ENR-ETP-GAT-CN-LEV-MEM-NA-NOR-S-SUZ-SXT-TE-W ^2^
SF (1)	FOX-CPD-CAZ-CRO-C-CIP-DO-ENR-ETP-GAT-CN-IPM-MEM-MH-NA-NOR-PRL-S-SUZ-SXT-TE-TOB-W ^2^
SG (1)	AMP-CIP-CT-DO-ENR-GAT-CN-LEV-NA-NOR-PRL-S-SUZ-SXT-TE-TOB-W
SH (1)	AMP-CIP-CT-DO-ENR-GAT-CN-LEV-MH-NA-NOR-S-SUZ-SXT-TE-W
SE (1)	AMP-CIP-CT-ENR-CN-LEV.NA-PRL-S-SUZ-SXT-TE-TOB-W
SC (1)	C-CIP-CT-DO-ENR-GAT-LEV-NA-NOR-S-SUZ-SXT-TE-W
SG (1)	AMP-SAM-CPD-CAZ-CRO-CIP-CT-DO-PRL-S-SUZ-SXT-TE-W
SC (1)	ATM-CPD-CT-DO-ENR-MH-NA-S-SUZ-SXT-TE-W
HB (1)	CIP-DO-ENR-ETP-MH-NA-S-SUZ-TE-TGC
SC (1)	ATM-DO-MR-S-SUZ-SXT-TE-TGC-W
SE (1)	DO-S-SUZ-SXT-TE-W
HB (1)	CT-DO-ENR-NA-S-TE

^1^ AK: amikacin, AUG: amoxicillin-clavulanate, AMP: ampicillin, SAM: ampicillin-surbactam, ATM: aztreonam, FEP: cefepime, CTX: cefotaxime, FOX: cefoxitin, CPD: cefpodoxime, CAZ: ceftazidime, CRO: ceftriaxone, C: chloramphenicol; CIP: ciprofloxacin, CT: colistin, DOR: doripenem, DO: doxycycline, ENR: enrofloxacin, ETP: ertapenem, GAT: gatifloxacin, CN: gentamicin, IPM: imipenem, LEV: levofloxacino, MEM: meropenem, MH: minocycline, NA: nalidixic acid, NOR: norfloxacin, PRL: piperacillin, S; streptomycin, SUZ: sulfadiazine, SXT: trimethoprim-sulfamethoxazole, TE: tetracycline, TGC: tigecycline, TOB: tobramycin, and W: trimethoprim. ^2^ ESBL-producing strain.

**Table 9:** Microbiological analysis: media, temperature, and incubation times.

Bacteria	Agar Media (Provider)	Conditions	
Mesophiles	Plate Count (Scharlau, Barcelona, Spain)	30 °C	48 h
Staphylococci	Mannitol Salt (Oxoid, Basingstoke, Hampshire, UK)	35 °C	36 h
*Enterobacterales*	MacConkey (Oxoid, Basingstoke, Hampshire, UK)	37 °C	24 h
Pseudomonas	Chromogenic for Pseudomonas (Scharlau, Barcelona, Spain)	30 °C	72 h
*Campylobacter* spp.	Brilliance Campy Count ^1^ (Oxoid, Basingstoke, Hampshire, UK)	42 °C	48 h
*Listeria monocytogenes*	ALOA (BioMérieux, Lyon, France)	30 °C	24 h
Methicillin-resistant *S. aureus*	ChromID MRSA (BioMérieux, Lyon, France)	37 °C	24 h
ESBL-producing *E. Coli*	ChromID ESBL (BioMérieux, Lyon, France)	37 °C	24 h

^1^ incubated under microaerobic conditions.

## Data Availability

Data are available upon request to authors.
